# Dietary Interventions for Short Bowel Syndrome in Adults

**DOI:** 10.3390/nu17132198

**Published:** 2025-07-01

**Authors:** Cassandra Pogatschnik, Lindsey Russell

**Affiliations:** 1 Center for Human Nutrition, Cleveland Clinic, Cleveland, OH 44195, USA; 2 Department of Gastroenterology, Hepatology, and Nutrition, Cleveland Clinic, Cleveland, OH 44195, USA; 3 Department of Medicine, Cleveland Clinic Lerner College of Medicine, Case Western Reserve University, Cleveland, OH 44195, USA

**Keywords:** short bowel syndrome, oral rehydration solution, dietary therapy, impaired hydration, nutritional support

## Abstract

Short bowel syndrome (SBS) is a rare but complex medical condition that requires expertise in management. The etiology in adults is commonly surgical resection for Crohn’s disease or mesenteric ischemia and is classified based on the anatomy of the remaining bowel. An accurate assessment of the anatomy and nutritional and hydration status is necessary. Dietary therapy is essential to induce adaptation in SBS, provide adequate nutritional needs, and manage symptoms including stool burden. As general SBS guidelines on nutritional support and dietary interventions exist, SBS is unique to the individual and nutrition must also be personalized to the individual to improve quality of life. This review will highlight the principles of adaptation, dietary interventions in SBS, as well as future directions for this field.

## 1. Introduction

Short bowel syndrome (SBS) is a rare and chronic malabsorptive disease caused by the surgical resection of the small bowel (SB) and occurs in 15% of adults undergoing intestinal resection [1]. Around 3 million people in the US and around 25,000 on home PN have SBS [2,3,4]. A recent study estimated the worldwide prevalence of 0.12–2.74 per 100,000 depending on the geographical region [5]. The etiology of SBS in adults is most commonly due to mesenteric ischemia, followed by Crohn’s disease [6], and malignancy [7]. SBS can lead to intestinal insufficiency due to a lack of absorptive capacity and amount of bowel remaining and can progress to chronic intestinal failure [8,9] needing additional support, such as enteral nutrition or intravenous (IV) nutrition or hydration. Patients with SBS have a lower quality of life than those without SBS and limiting days on IV support led to a higher quality of life [10]. This highlights the need for multidisciplinary care to wean patients from IV support with medications to slow down intestinal losses and increase absorptive capacity while pairing it with proper dietary intake.

**Figure 1:**
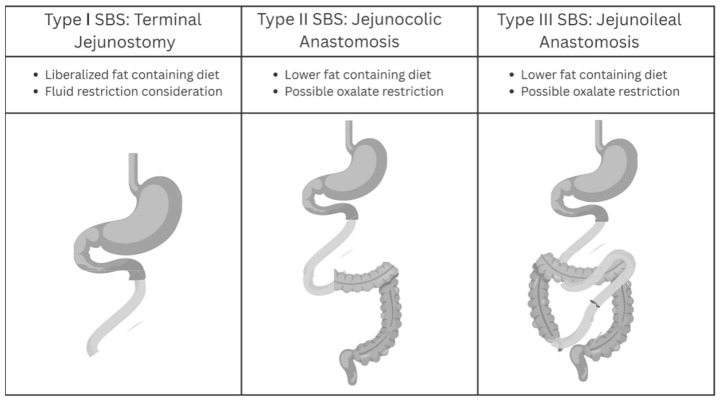
Classification of SBS.

There are three main types of SBS, defined by anatomy: Type I includes end jejunostomy, Type II involves jejunal ileal resection with a jejunal colonic anastomosis, and Type III is classified as jejunal ileal anastomosis with an intact ileocecal valve and entire colon [10,11,12] (see Figure 1).

Intestinal rehabilitation or the promotion of intestinal adaptation is requisite for the management of SBS. Oral nutrition is instrumental in the treatment of SBS, with enteral autonomy being the goal in intestinal adaptation. Although all forms of nutrition support can be utilized to provide adequate nutrition, the use of short-bowel oral diet strategies should not be ignored and can promote intestinal adaptation, improve absorption, and preserve hydration and nutritional status [13,14]. The introduction of oral or enteral nutrition should be used if the gut appears functional and the patient’s feculent affluent or output is less than 800 milliliters while nil per os [13].

This review will briefly touch on adaptation and the role of dietary therapy in the management of patients with SBS, both with inclusive advice applicable to all patients with SBS as well as specific dietary advice catering to specific SBS anatomy types. A thorough search of recent literature through the Medline and Embase databases using the search terms “Diet” or “Diet interventions” with “Short Bowel” (see Supplemental Figure S1) was performed. We focused on the latest literature available; however, some older references were included if important concepts were highlighted. We focused on the management of adults, however, literature focusing on pediatrics with applicable advice was included. In addition, recent guidelines were reviewed. In the absence of evidence, recommendations reflect the authors’ expert opinion. Notably, the majority of the literature about the effect of diet on SBS management was conducted on a limited number of patients, and the most recent ESPEN guideline dietary advice on chronic intestinal failure is based on low-grade evidence [15].

## 2. Adaptation and How Anatomy Impacts Absorption

Intestinal adaptation is a natural process that starts post-operatively and continues two to three years post-intestinal resection [13,16]. Structural and functional changes, including increases in villous height, increases in crypt depth, and increases in bowel dilation, increase the intestinal surface area without increasing the actual SB length [17,18]. Liver, pancreatic, and stomach functions also influence the intestine’s ability to adapt to the stimulus of intestinal secretions/bile intraluminally [19]. In addition, gut hormones serve as trophic factors, such as GLP-1 and GLP-2, and peptide YY is thought to be involved in a hormonal “break” in adapted bowels to slow down transit time and allow for the absorption of nutrients [19,20,21]. A preclinical model found that administering fecal contents from a patient with SBS to germ-free mice led to increased circulation of Ghrelin and GLP-1, suggesting the role of microbiota in gut hormone signaling [21].

Enteral nutrients are essential for the induction of adaptation due to direct contact with the epithelial cells. This contact stimulates mucosal hyperplasia, stimulating the production of trophic pancreatic-biliary secretions [17,22,23], and increased blood flow to the gut with feeding [19]. Diligent diet adherence is warranted initially but may be loosened as the adaptation period evolves and adjusts based on the patient’s needs and preferences [24]. Individual anatomy impacts the therapeutic diet requirements as outlined in the next sections. In addition, monitoring for intestinal mucosal integrity is important, given that Crohn’s disease is the most common cause of SBS, ongoing evaluation for active disease affecting absorption is necessary. Finally, the portion of SB remaining predicts adaptation capabilities, as a study in a rat model found that with proximal SB resection, the distal ileum had greater adaptation capability compared to when the distal SB was resected [25].

It is important to clarify the anatomy and length of the bowel that is preserved as it predicts the ability to wean off IV support, the need for specific micronutrient supplementation, and the probability of adaptation. Many surgeons do report resection length and estimate the length of the remaining bowel intraoperative, but it can be challenging at times due to adhesions or scar tissue to obtain an accurate measurement. Pathology specimens can be used to assess the length of bowel resected; however, it is not always available. A recent systematic review compared different imaging modalities to pathologic specimens in estimating bowel length [26]. Notably, SB follow-through with X-ray led to a significant underestimation of bowel length and that 3D imaging, such as CT or MRI, had a better prediction of bowel length. However, there are challenges for availability and feasibility [26]. Others have described the use of plasma citrulline levels as a tentative marker of bowel absorption capacity of the remaining bowel, suggesting using certain cut-offs to help predict enteral autonomy [27].

Knowing the portion of SB resected can help predict needs, (absorption, etc.) due to the specific absorption capabilities of each section of the small intestine [11,28]. For optimal intestinal absorption, the gastrointestinal tract (GI) must absorb 7 to 9 L of fluid secreted by itself along with 2–3 L of enteral intake, which is limited with anatomical alteration [11]. It was determined in a prior study that intestinal failure patients with SB resection who avoided home parenteral nutrition (PN) had wet weight (difference between the weight of ingested food and excrement) that was generally >1.4 kg/day and absorption of 84% of predicted needs as measured by Harris–Benedict (approximately 1200 kcal a day), suggesting the use of wet weight as a predictor of intestinal failure [29].

The presence of the colon in continuity generally has a better prognosis and dietary management of patients with SBS. Prior studies have noted that even with 50 cm of SB remaining, the ileocecal valve (ICV) and entire colon are sufficient to maintain hydration status and the ability to wean off nutritional support [30].

There are pharmaceutical and surgical treatments that can be offered to patients to help with intestinal adaptation and weaning off IV nutrition support. Antidiarrheals are commonly used to help slow down gut transit time. However, they must be timed before meals and oral intake to maximize their effects [24]. Synthetic gut hormones, such as GLP-2, are used to help initiate villous growth and wean IV support [1,19]. Case reports have specifically highlighted GLP-2’s effectiveness on stalled weaning efforts, often resulting in full enteral autonomy, suggesting positive effects even years after the initial resection [31]. Surgical options including various techniques to lengthen the bowel or small bowel transplantations are reviewed elsewhere [1,32].

## 3. General Short Bowel Syndrome Diet Suggestions and Strategies

### 3.1. Hyperphagia

It is generally accepted that patients with SBS will have hyperphagia, defined as eating at least 1.5× of predictive resting energy expenditure [33]. A prior study noted that at least 83% of a large cohort of patients with SBS had hyperphagia. However, patients with Type II/III SBS had significantly higher rates of hyperphagia, further questioning the role of colonic microbiota in this behavior [34]. It is generally recommended for patients to have multiple small meals per day and to separate liquids and solids [15]. Patients with infant-onset intestinal failure may have limited early oral feeding attempts due to prematurity complications, oral aversion (such as early intubation), and GI symptoms (pain, vomiting, distention, aspiration, and diarrhea), leading to adverse experiences with food and the development of feeding disorders [35]). These patients, along with patients who have acquired SBS later in life, may have reduced appetites or food aversions, where clinical strategies to improve oral intake may include multidisciplinary therapies centered on the relationship with food, the psychology around the gut–brain axis, and appetite stimulants. Alarms or consistent visual cues to create improved eating habits may be beneficial in incorporating dietary recommendations for multiple meals per day [36].

### 3.2. Macronutrient Needs

Patients with SBS generally should consume all three macronutrients, protein, carbohydrates, and fat daily (Table 1) [8]. All intraluminal nutrients stimulate proliferation of gut epithelial cells, the production of trophic hormones, and stimulate other GI organ secretion, and whole foods are recommended [15]. Recommended protein needs are similar between the SBS types as amino acids and peptides are efficiently absorbed in the SB [2,37]. Total proteins are preferred for intestinal adaptation, as the more complex the digestion of a nutrient, the greater hyperphagia ensues [38]. Protein with high bioavailability should be encouraged [39]. High-protein needs may be needed postoperatively to mitigate muscle loss initially.

Carbohydrates are most readily used in patients with SBS [37,39]. Generally, complex carbohydrates are preferred over simple high-sugary food items such as juice or candy, which can lead to osmotic diarrhea [37,39]. Pairing carbohydrates with protein for each meal can help with slowing gastric emptying and bowel transit. Patients with documented small bacterial overgrowth may benefit from limiting carbohydrates in Type II/III SBS [23].

Fat malabsorption is a hallmark of SBS due to lack of absorptive capacity of the remaining bowel, impaired motility, alterations in pancreatic secretions, and impaired bile acid circulation [2]. Medium-chain fatty acids (MCT), notably, can be absorbed directly by enterocytes and may be an alternative in patients with SBS [2,24,37]. However, it should be noted that MCT will not supply essential fatty acids, and long-chain triglycerides will be needed to prevent essential fatty acid deficiency [40]. Although prior studies did not show the differences in oral pancreatic enzyme replacement in fat malabsorption for patients with SBS [41], a recent study assessed the utility of lipase cartilages for patients with SBS on tube feeds and found better tolerance [42]. Details on fat recommendations are highlighted in the next section.

### 3.3. Oral Rehydration Therapy

In addition to ensuring adequate caloric intake, it is key to establish the hydration status for patients to avoid dehydration and to ensure patients with SBS are drinking the correct fluid [18]. Hypertonic solutions will lead to osmotic diarrhea, and hypotonic solutions may lead to loss of electrolytes and increased effluent [43]. As water absorption in the small bowel relies on sodium-glucose cotransporter 2 (SGLT2) receptors, oral rehydration solution (ORS) is needed to help increase water net absorption, improve diarrhea, and overall output [43]. There are many commercial products, however, patients should avoid the sugar-free versions as they lack the necessary glucose for SGLT2-mediated absorption. Although the commercial products are convenient, they can become expensive over time, especially if patients are required to drink liters per day. It may be advantageous for patients to make ORS themselves with flavoring (Table 2). Education is needed for patients, families, and other healthcare professionals who specifically suggest that “drinking more water” is detrimental to SBS, and ORS is warranted to help meet hydration needs.

### 3.4. Micronutrients and Vitafmins

The area of resected bowel can influence the need to supplement specific micronutrients and vitamins. Classically, Vitamin B12 needs supplementation if the majority of the ileum is resected due to absorption patterns [11]. With jejunal resections, it is unique to each patient in determining what support they may need, and it is recommended to measure a full micronutrient panel initially (Vitamin A, B1 B12, C, D, E, Folate, Iron, Zinc, Copper, Selenium) and correct the abnormal levels [44]. Due to the absorptive capacity of SBS, higher dosages of supplementations are needed compared to patients without SBS [44]. In addition, attentiveness to supplement formulation is key to avoiding diarrhea exacerbation as some liquid supplements contain sorbitol and salt preparations, such as magnesium oxide, which may lead to diarrhea more so than another formulation, such as magnesium bisglycinate [45].

### 3.5. Soluble Fiber

Soluble fiber is generally preferred in SBS compared to insoluble fiber. Soluble fiber can bulk up the stool and help limiting the effluent output in stomas and help ferment into carbohydrates within the colon [46,47]. Soluble fiber can be viscous forming, leading to increased intestinal transit time, and different varieties have fermentable properties, leading to short-chain fatty acid production [18,46,47]. However, soluble fiber can also be detrimental as it can retain water and nutrients, leading to a lack of mucosal absorption or fermentable to the point of creating gas and bloating for patients [46]. A survey of practitioners managing SBS noted that the most commonly prescribed soluble fiber was pectin at (62.8%), and the two most common reasons to initiate soluble fiber in SBS were stool consistency and volume of stool output [46]. However, many providers noted that soluble fiber was held due to bloating in many cases [46]. More studies are needed to assess the utility of soluble fiber in the setting of SBS as current guidelines suggest against the use of soluble fiber, but they are based on poor-quality evidence [15].

### 3.6. Use of Nutrition Support to Supplement PO Intake

Patients with SBS in the adaptive phase may need parenteral support to help supplement their oral intake to ensure they maintain their nutritional needs. Especially in the setting of major abdominal surgery, adequate protein and nutrition are vital to ensure proper wound healing. Individuals with <200 cm of the small intestine to an ostomy generally do require specialized nutrient and fluid supplementation, with many requiring nutrition support long-term post insult or injury [8,11].

The use of a multidisciplinary team is necessary to arrange for parenteral nutrition both in the hospital, as well as in the outpatient setting [3,4,44,48]. Due to the dynamic needs of SBS patients during the first two years, consistent follow-up with a reassessment of needs and nutritional status is vital to balance nutritional needs and weaning to obtain enteral autonomy [15,39].

## 4. Specific Diet Therapies by Anatomy

### 4.1. Type I SBS

Generally, Type 1 SBS leads to increased fluid losses and special attention to hydration status is necessary, where many require IV support [18,37,39]. Patients who exhibit intestinal output of three liters with jejunal lengths <65 cm should limit their oral intake and restrict their fluid intake to <1.5 L for feeding pleasure only [13]. A study by Sobocki et al. compared two small cohorts with end jejunostomies for a six-month duration, one group was to restrict orals in an effort to keep their enteric output <1000 mL (noted 5 out of 10 patients remained NPO for the study duration), and an alternate group was left unrestricted [49]. The results demonstrated that patients left unrestricted had a lower quality of life, emptying stoma bags 2–5 per night, versus 0 in the restricted group, and suffered stoma leak, stoma skin erosion, increased hunger, and thirst (along with increased hepatic and renal dysfunction and required increased episodes of rehospitalization [49]). It was concluded that because oral feeding causes more losses of nutrients and fluid, a patient is likely frustrated as the meal is less satisfying, and the consequences of oral intake are high [49]. A case study published by Almperti et al. further demonstrated PN weaning success in a patient with Type I SBS, 80 cm to jejunostomy. This was completed first with a tightly restricted diet (1000–1200 kcal/day, ORS (500 mL/day), limited water intake (<500 mL/day) preventing “net secretion”, with PN wean of 50% total caloric provisions [50]. As output stabilized, GLP-2 was initiated at 1 mg/kg/day with diet liberalization to an unrestricted diet. This resulted in full PN wean and stoma output remaining stable at 1.3–1.5 L/day [50]. Another recent cross-over study in 10 patients demonstrated that a semi-elemental oral nutritional supplement led to higher fecal weight and sodium loss than a polymeric formula [51]. Prior studies have shown that diets high in dietary fat showed no difference in fecal fat composition and effluent volume [8,52]. Therefore, it is thought that patients with Type I anatomy benefit from a diet richer in fats than other SBS anatomies. Dietary recommendations include a moderate fat content of 30–40% of total caloric intake [13,18,53], as diets higher in fat content generate a lower net osmolality that favors absorption [11,39].

### 4.2. Type II & III SBS

Patients with anatomical configuration with a colon in continuity benefit from increased fluid absorption and can salvage up to 1000 calories per day due to bacterial fermentation of unabsorbed carbohydrates, resulting in absorbable short-chain fatty acids [54]). Therefore, these anatomies that lack the ICV require both a diet richer in complex carbohydrates and lower in fat content due to fat malabsorption, steatorrhea, and consideration for bile acid wasting [2,37]. Notably, a prior cross-over study noted that patients with SBS with a colon in continuity on a high carbohydrate/low fat diet had a reduction in stool output compared to a high fat/low carbohydrate diet [47]. Mal-absorbed long-chain fatty acids (LCFA) decrease fluid and sodium absorption and can inhibit carbohydrate fermentation. Further, LCFAs bind calcium and magnesium salts, increasing stool loss and promoting oxalate absorption by the colon [40,55]. However, it is noted that limiting fat may be disadvantageous as little impact may be noted in the volume of enteric losses, increase the risk for essential fatty acid deficiency, and hinder the promotion of adequate caloric intake and hyperphagia [55].

Metabolic acidosis from D-Lactic acid is a rare complication of SBS, leading to vague symptoms such as confusion, ataxia, and irritability due to the fermentation of carbohydrates in the colon [15,56,57]. It is confirmed by the blood concentration of D isomer of lactate and treated with antibiotics, simple carbohydrate avoidance, and careful reintroduction of complex carbohydrates [56,57]

Oxalate from the diet is not absorbed in the SB, and in normal GI anatomy, once in the colon, forms a complex with calcium for secretion via stool. However, in Type II/III sub-types of SBS, there is a high risk of unbound oxalate being absorbed in the colon, leading to oxalate stones, especially in patients with kidney dysfunction [18,29,39]. There have been other case reports of hyperoxaluria, leading to deposition into other tissues such as bone marrow, thyroid, and blood vessels in patients with SBS [58]. A low oxalate diet is recommended for patients with known oxalate stones or kidney dysfunction [39]. Treatment with adequate calcium supplementation, typically 500 mg with meals, can help chelate oxalate in the colon and prevent oxaluria [59].

## 5. Future Directions

There have been limited recent trials on diet composition and SBS management. A recent study that evaluated clinical practices through surveys across various SBS expert centers demonstrated that the dietary management of patients greatly varied with Type II and Type III SBS compared to Type I [60]. There may be a role for dietary intake records and recommendations in clinical trials assessing new drugs for the medical management of SBS to ensure diet is not a major confounding factor. This also highlights the opportunity to pool data across expert centers as this is a rare condition to compare and inform guidelines for improving patient outcomes. A recent study found that a high-polyamine diet in a mouse model of SBS led to an increase in villous length in the adaptation phase and mitigated inflammation in the liver, which suggests a potential role of polyamines in the treatment of SBS [61].

The influence of the microbiome on intestinal adaptation and hyperphagia patterns in patients with SBS needs further exploration for implications in clinical practice [20,34,62]. Microbiota composition in patients with SBS is distinct from healthy controls, with an unbalanced and high prevalence of *Lactobacillus* and poor diversity of *Bacteroidetes* and *Clostridium coccoides* [62]. Other functional studies have shown more efficient nutrient recovery in the microbiota of SBS compared to controls [20]. In addition, the microbiota in SBS had the ability to induce higher levels of Ghrelin and GLP-1 when transplanted into germ-free mice [21], highlighting the cross-talk between the microbiome and gut hormones. Another study assessed the influence on the microbiota composition for bile acid metabolism and the effect of whole food consumption in 55 children with SBS [63]. Interestingly, further studies regarding the utilization of probiotics or fermented dairy products with varying bacterial isolates and concentrations are warranted for calcium oxalate deformation as oxalates have been found to be degradable by bacterial species such as *Oxlobactor formigenes*, *Enterococcus faecaleis*, *Lactobacillus acidophilus*, and *Eubacterium lentum* [64]. Reduction of oxalate reabsorption would be instrumental in reducing the incidence of calcium oxalate formation in patients with preserved colon anatomies [64]. Further exploration of the microbiota and manipulation of such in SBS to avoid complications, such as D-Lactate acidosis and oxaluria, and help wean nutritional support and gain enteral autonomy is needed. As more adults are surviving with SBS and developing other chronic conditions such as hypertension or chronic kidney disease, collaboration across specialties is warranted to ensure consistent communication is made with the patient. It can be challenging when patients are told to avoid high salt for cardiovascular health but encouraged ORS with salt to maintain hydration. Finally, objective measurements of metabolic rates through indirect calorimetry and body composition can be used to better assess the nutritional needs of patients with SBS. Patients with a history of obesity and bariatric surgery (leading to internal hernias) and bowel resections (leading to SBS), who have achieved intestinal autonomy, are especially challenging to provide care for due to prior metabolism alterations and body composition, and to balance their metabolic status with the limited absorptive capacity in SBS and limitations in predictive equations for nutritional needs in patients with obesity. More studies are needed to assess patient outcomes in these specific patients.

## 6. Conclusions

Overall, SBS represents a rare but complex medical condition that requires a multidisciplinary team to effectively manage these patients. Diet is essential in SBS to help initiate and maintain intestinal adaptation, manage stool burden, and maintain nutritional status to wean off IV support. Patients need to be monitored closely with assessing hydration status and nutritional assessments to ensure the nutrition plan is optimized, especially throughout the adaptation phase as nutritional needs may change. However, the importance of oral dietary advice is vital for success. As SBS is a chronic condition, patients generally become their own experts on which foods work best for them, and an individualized nutrition plan is essential to cater to their unique metabolic and cultural needs. Dietary adjustments and provisions are essential to maintain health in SBS. More studies are needed to determine the influence of diet on the efficacy of medical treatments and long-term outcomes in patients with SBS.

## Figures and Tables

**Table 1:** Summary of dietary recommendations for SBS [18,37,39].

**General Recommendations**	
General	Multiple small meals a day Separate liquids and solids ORS for liquid intake Lactose-containing foods if tolerated Consider soluble fiber if tolerated
Macronutrients	High-quality protein throughout the day Limit concentrated sweets (juice/desserts) Complex carbohydrates with meals
Micronutrients	Consider anatomy for risk of specific micronutrient deficiency (ex. Vitamin B12) Measure micronutrient panel (Vitamin A, B1 B12, C, D, E, Folate, Iron, Zinc, Copper, Selenium) and supplement accordingly Monitor and replace magnesium
**Specific Recommendations**	
End jejunostomy (Type 1)	Monitor for steatorrhea and adjust oral fat intake Consider MCT oil 1-3Tbsp/day spread out
Colon in continuity (Type 2/3)	Monitor for steatorrhea and bile acid diarrhea and adjust fat in diet accordingly Consider low oxalate diet if impaired kidney function or known oxalate stones

**Table 2:** Standard oral rehydration recipes.

Recipe for Homemade ORS	
4 cups water	¾ tsp salt
2 Tbsp sugar	Flavoring as needed for taste

## Supplementary Materials

The following supporting information can be downloaded at: https://www.mdpi.com/article/10.3390/nu17132198/s1, Figure S1. Medline and Embase database search PRISMA diagram.
