# Supplementary material for: Dietary Interventions for Short Bowel Syndrome in Adults
Source: Nutrients. 2025 Jul 1;17(13):2198. doi: 10.3390/nu17132198 (PMC12251760; doi:10.3390/nu17132198)

Supplemental Figure S1. Medline and Embase database search PRISMA diagram.

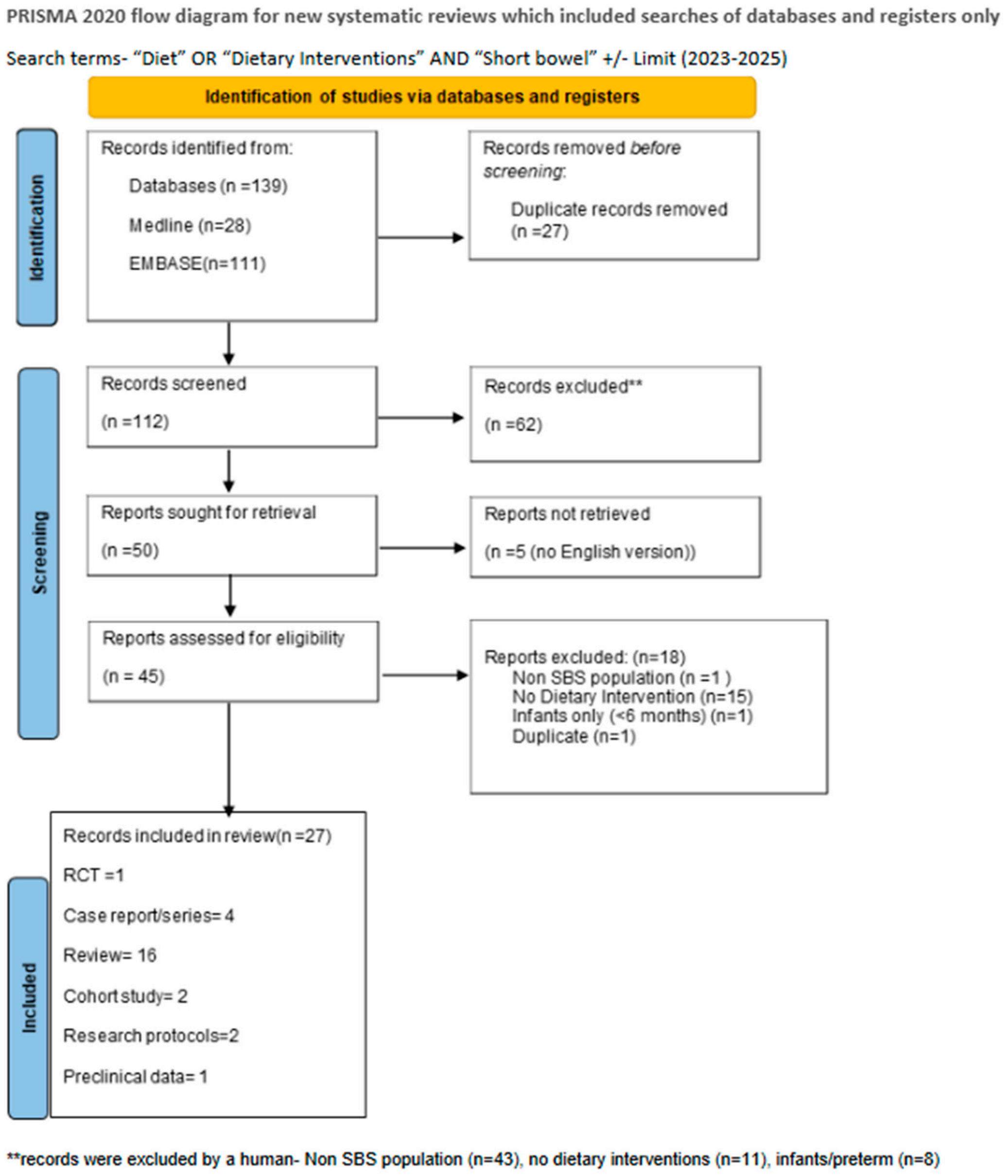

Supplement: Supplementary file 1 [file nutrients-17-02198-s001.zip › nutrients-3692207-supplementary.pdf]
